# Author Correction: Uplift of the central transantarctic mountains

**DOI:** 10.1038/s41467-018-03349-y

**Published:** 2018-02-16

**Authors:** Phil Wannamaker, Graham Hill, John Stodt, Virginie Maris, Yasuo Ogawa, Kate Selway, Goran Boren, Edward Bertrand, Daniel Uhlmann, Bridget Ayling, A. Marie Green, Daniel Feucht

**Affiliations:** 1 University of Utah/Energy & Geoscience Institute, Salt Lake City, UT 84108 USA; 2 University of Canterbury, Gateway Antarctica, 8041 Christchurch, New Zealand; 3 Antarctica Scientific Ltd, 6011 Wellington, New Zealand; 4 Formerly at GNS Science, Natural Hazards Division, 5011 Lower Hutt, New Zealand; 5 Numeric Resources LLC, Salt Lake City, UT 84108 USA; 6 Tokyo Institute of Technology, Volcanic Fluid Research Center, 152-8550 Tokyo, Japan; 7 Department of Earth and Planetary Sciences, Macquarie University, Sydney, NSW 2109 Australia; 8 Department of Geology & Geophysics, University of Adelaide, Adelaide, SA 5005 Australia; 9 GNS Science, Natural Hazards Division, 5011 Lower Hutt, New Zealand; 10 First Light Mountain Guides, 74400 Chamonix, France; 11 Great Basin Center for Geothermal Energy, University of Nevada, Reno, NV 89557 USA; 12 Department of Geology & Geophysics, University of Utah, Salt Lake City, UT 84112 USA; 13 Department of Geological Sciences, University of Colorado, Boulder, CO 80309 USA

Correction to: *Nature Communications* 10.1038/s41467-017-01577-2, published online 17 November 2017

The original version of this Article incorrectly referenced the Figures in the Supplementary Information. References in the main Article to Supplementary Figure 7 through to Supplementary Figure 20 were previously incorrectly cited as Supplementary Figure 5 through to Supplementary Figure 18, respectively. This has now been corrected in both the PDF and HTML versions of the Article.

