# Supplementary material for: Uplift of the central transantarctic mountains
Source: Nat Commun. 2017 Nov 17;8:1588. doi: 10.1038/s41467-017-01577-2 (PMC5693935; doi:10.1038/s41467-017-01577-2)
Supplement: Supplementary file 1 — Supplementary Information [file 41467_2017_1577_MOESM1_ESM.pdf]

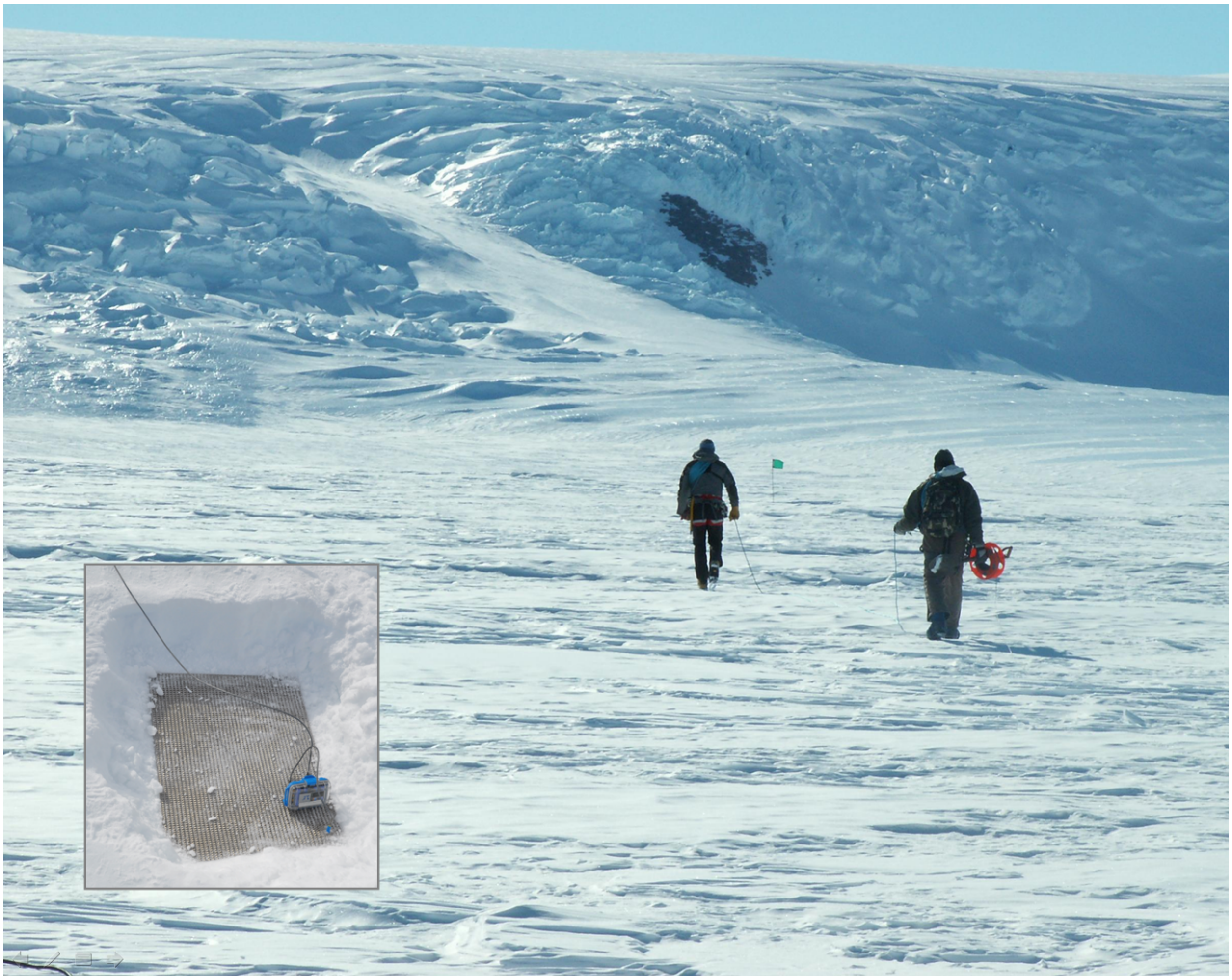

Supplementary Figure 1.

Harnessed, roped, paired travel to install E-field electrode at nominal 150 m spans in four orthogonal directions from MT site center. J. Pierce (left) and J. Stodt (right) pictured. Note green flag emplaced from prior recon/safety visit. Inset shows titanium sheet metal electrode and high-impedance buffer preamplifier just prior to burial. Photos by P. Wannamaker.

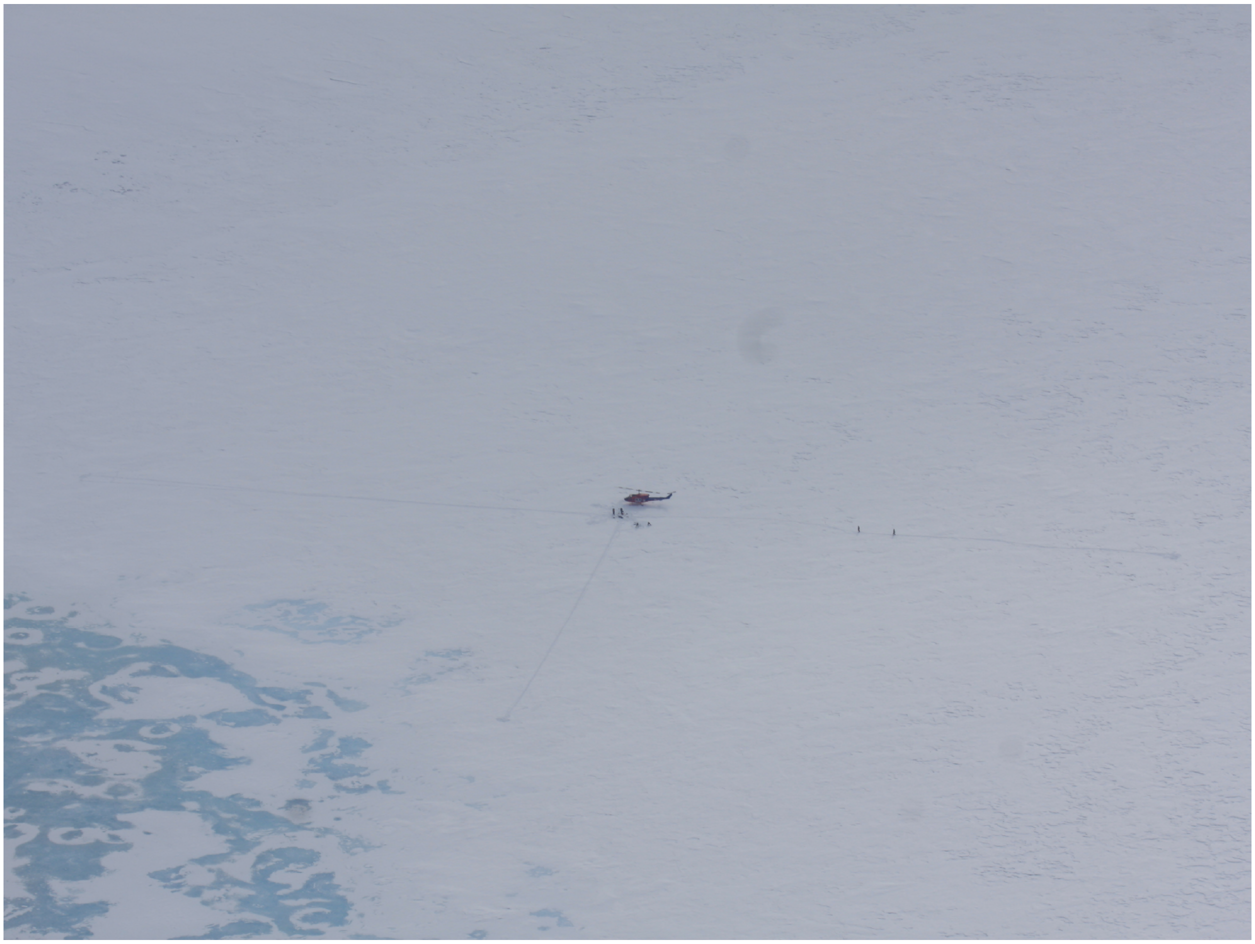

Supplementary Figure 2.

Helicopter view of site center by northeast boundary of RIS near completion of installation. Three of four electrode legs are completed. Site recording was unattended. Photo by J. Schas.

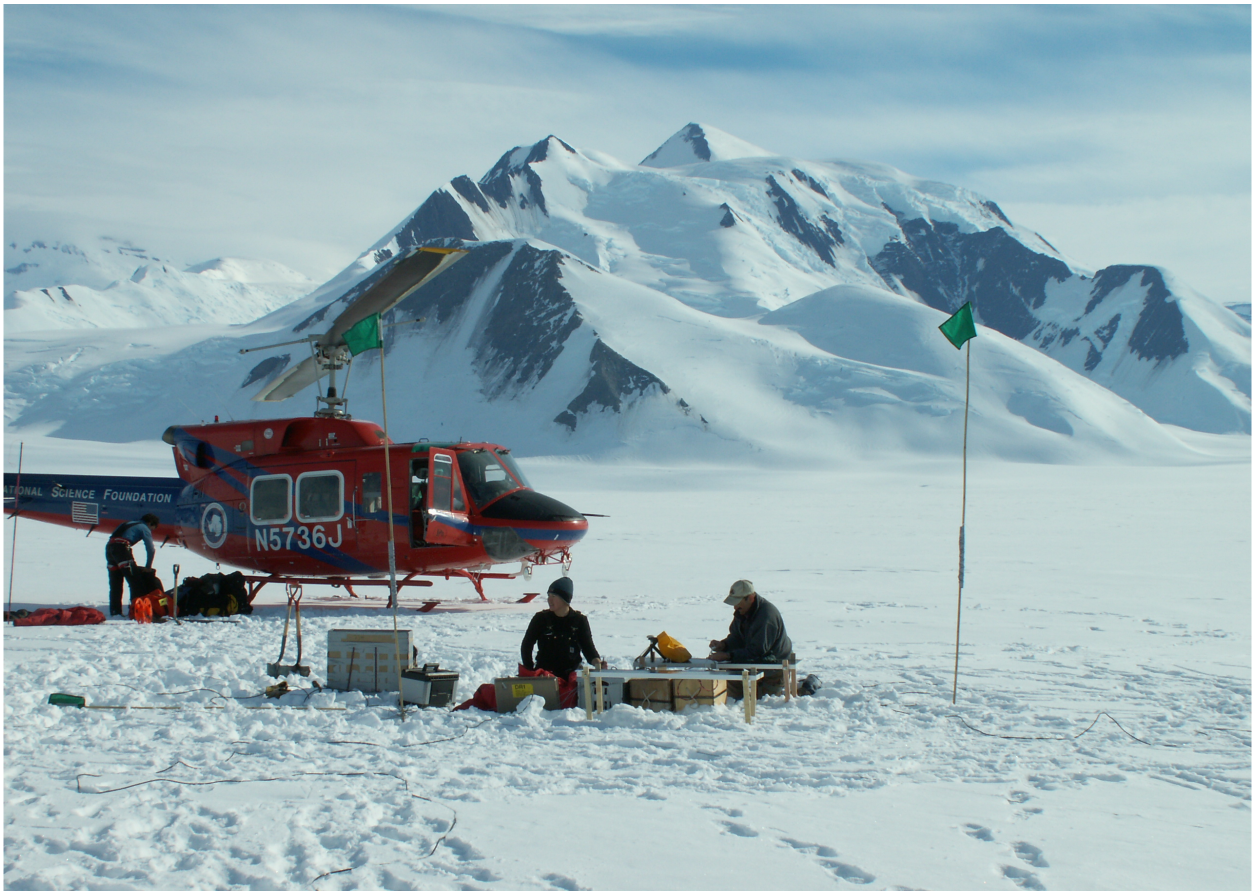

Supplementary Figure 3.

Ground view of site center by northeast boundary of RIS near completion of installation. J. Stodt (right), V. Maris (center) and D. Uhlmann (left) pictured. Site recording was unattended. Photo by P. Wannamaker.

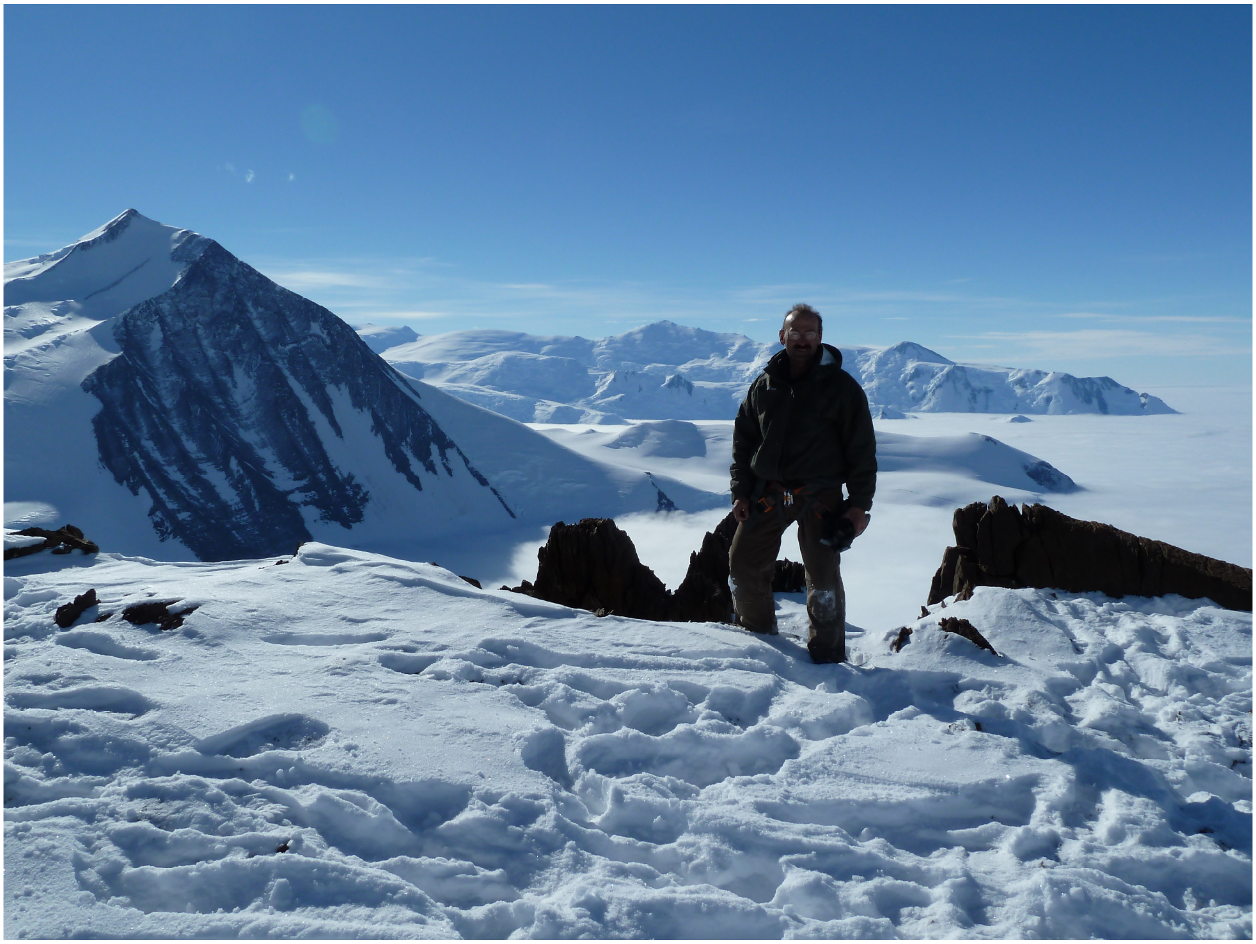

Supplementary Figure 4.

View grid southeastward along the TAM rangefront where our MT transect crosses it, ~400 m southeast of nearby MT site A06 (see Fig. 1). Fog ceiling is ~700 m above RIS surface. Personnel pictured is co-author Dr. John Stodt. Photo by P. Wannamaker.

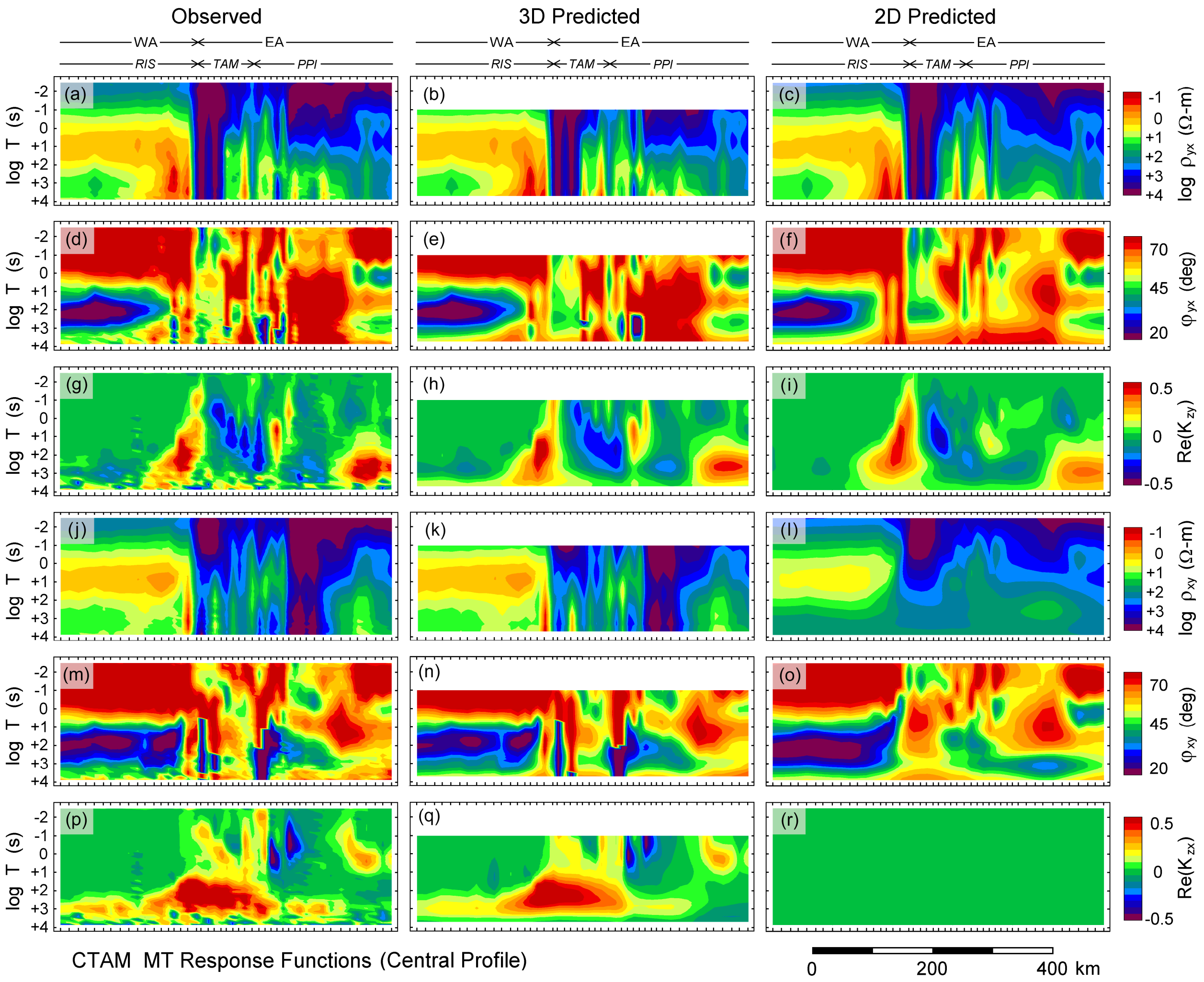

Supplementary Figure 5.

Pseudosections of primary MT observations plotted with a spectral colour scheme. For full description, see discussion around Fig. 4 in main text.

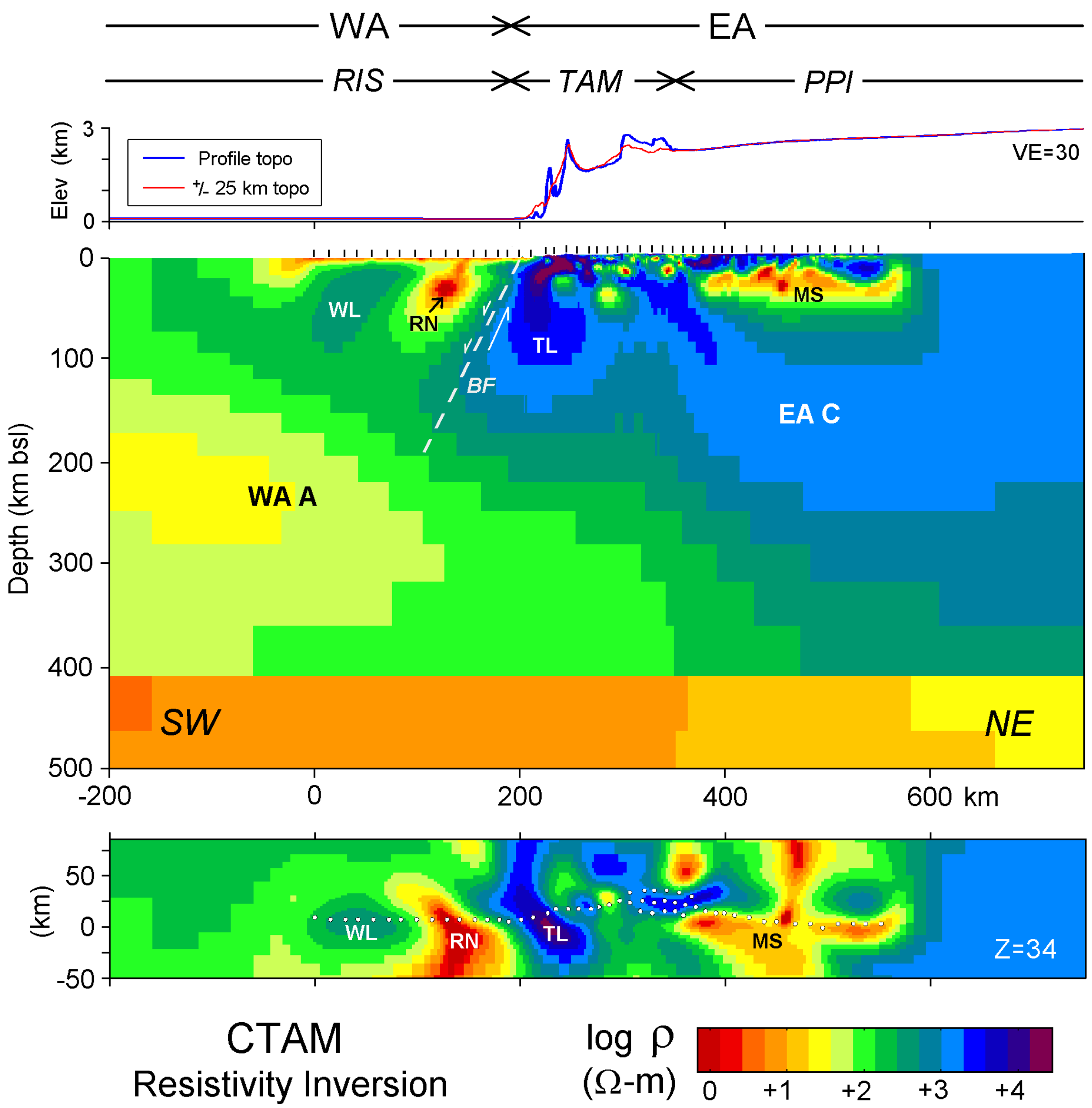

Supplementary Figure 6.

Three-dimensional resistivity inversion model for the central TAM plotted with a spectral colour scheme. For full description, see discussion around Fig. 6 in main text.

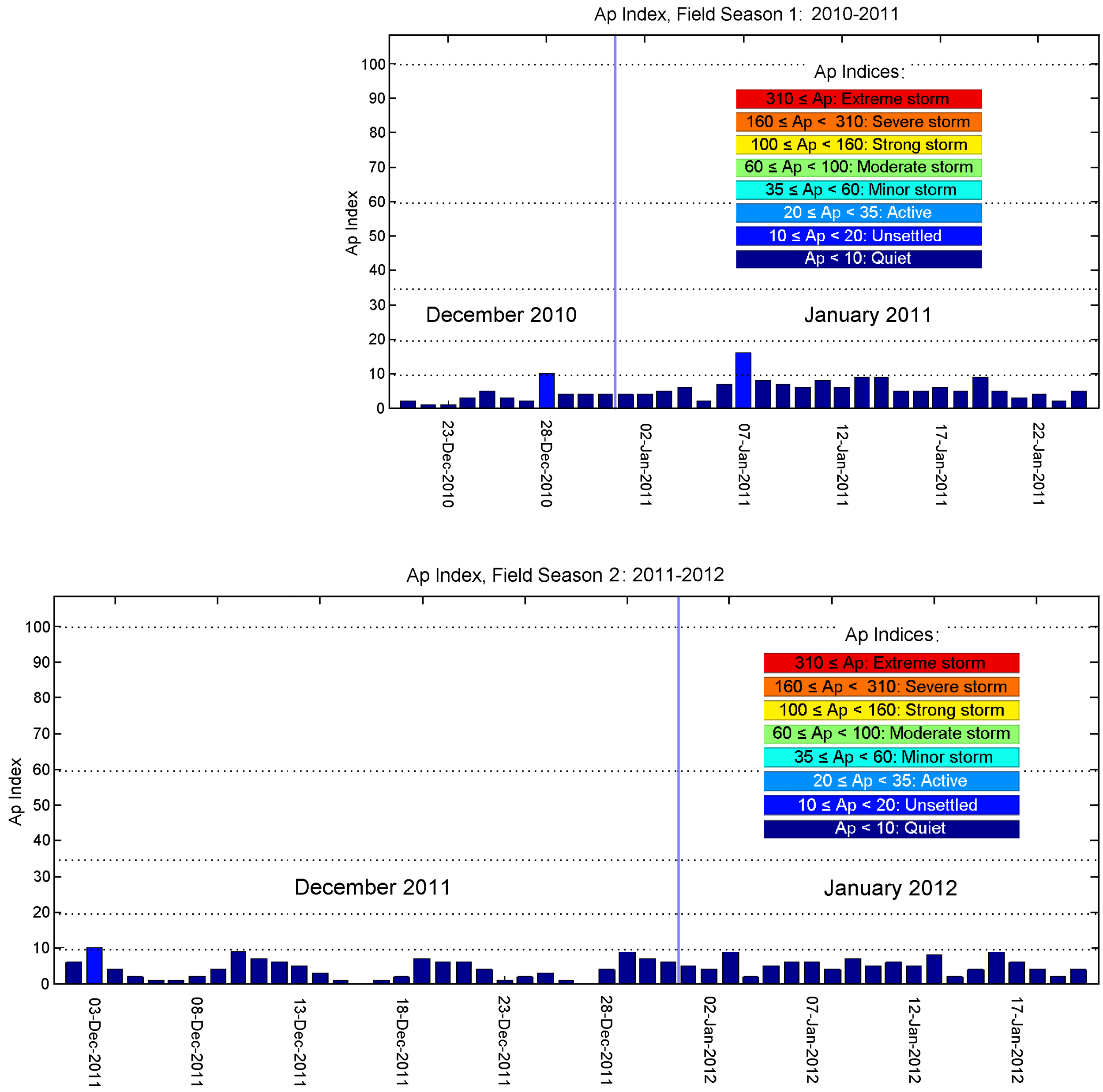

Supplementary Figure 7

Daily Ap magnetic activity indices over the duration of the two CTAM MT field seasons. Index values obtained from NOAA National Center for Environmental Information site[62]. Activity categories (e.g., "Active", etc) are from NOAA users guide to Forecast of Solar Geophysical Data[63].

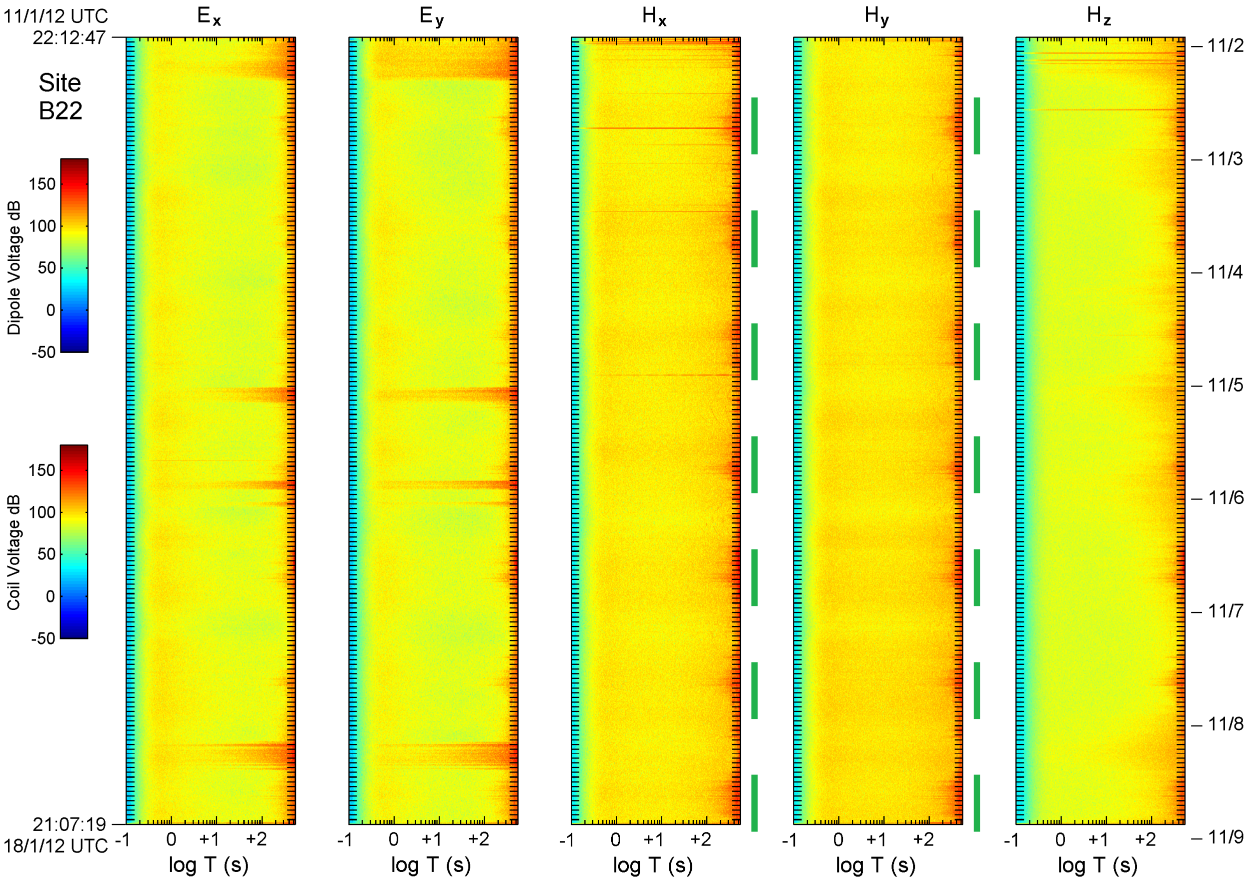

Supplementary Figure 8

MT signal spectral intensity versus time for the seven day recording interval of January 11 through January 18, 2012 at site B22 on the polar plateau. Diurnal S_q^p variation[64] is most evident in horizontal magnetic fields where the selected higher intensity 12 hour intervals are demarked by green bars. Occasional protracted windbursts are evident in the electric channels. Spectral calculations performed using the signal processing toolbox in Matlab ®.

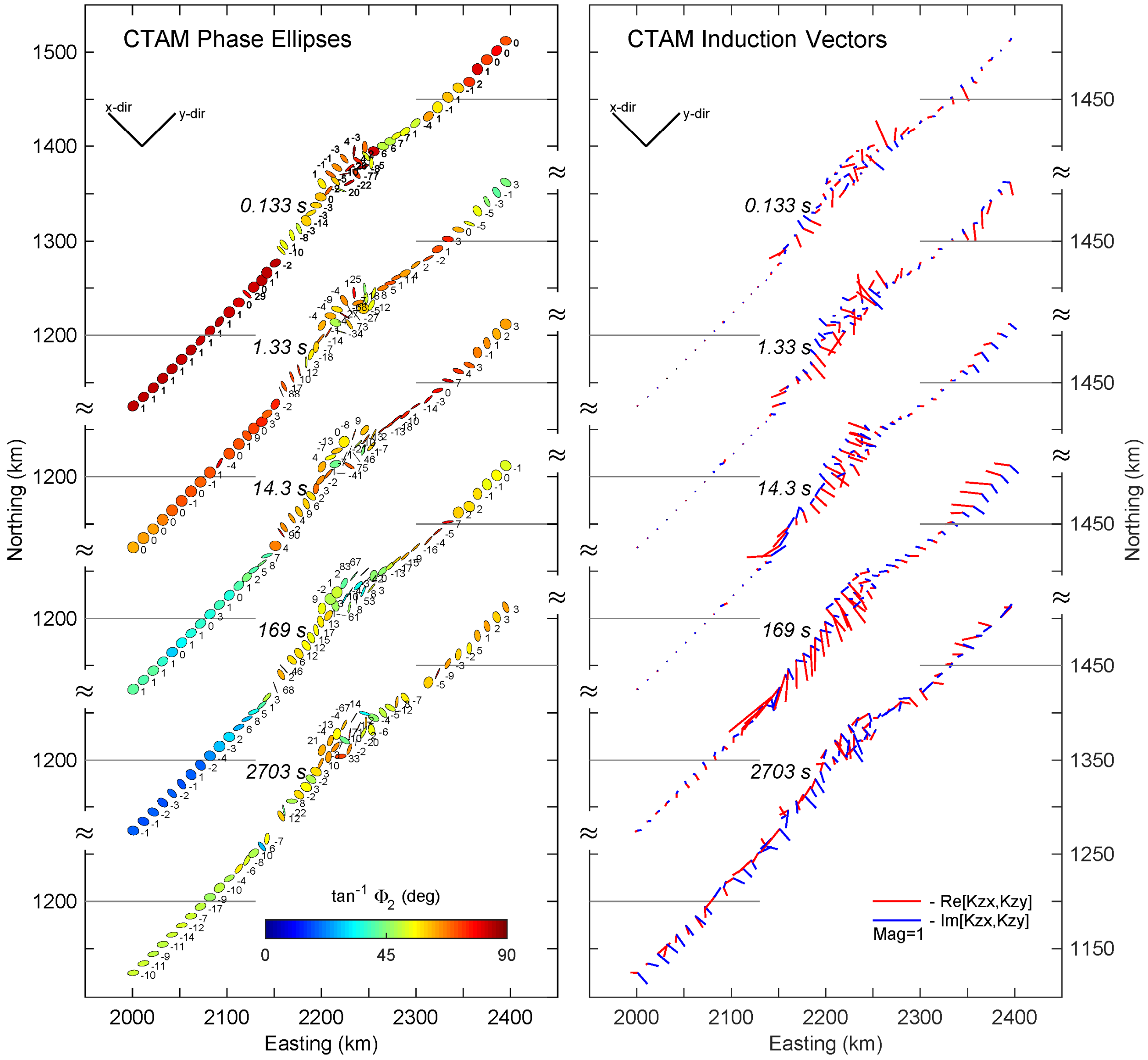

Supplementary Figure 9

Left (a): Normalized impedance phase ellipses at five periods for sites along the central Transantarctic Mountains MT transect. Ellipse color is phase invariant Φ_2[66,67]. Phase tensor skew values are printed beside each ellipse. Right (b): Induction vectors at the same five periods for sites along the central Transantarctic Mountains MT transect. Reversed convention used such that real vectors point toward conductors. Frequency plots are shifted 100 km southward from each other arbitrarily, and Polar Grid 100 km marks are labelled along sides (see Fig. 1). Assumed x and y axes for data coordinates shown to upper left.

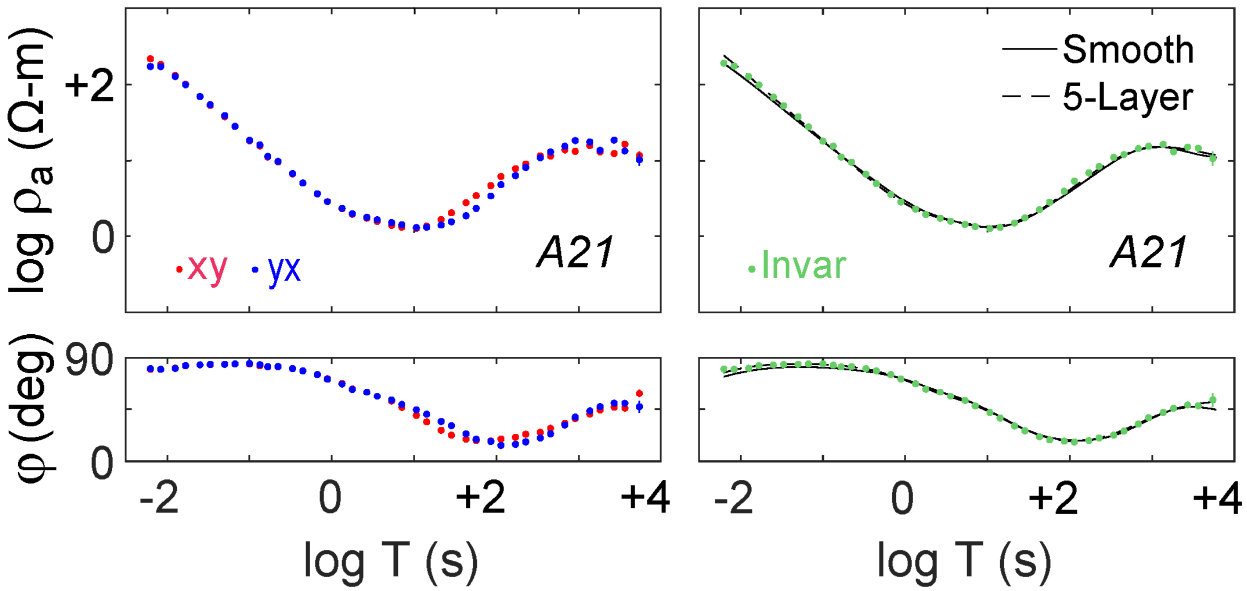

Supplementary Figure 10

Left: Off-diagonal apparent resistivities and impedance phases at Ross Ice Shelf (RIS) site A21 showing nearly isotropic behavior. Right: Impedance invariant apparent resistivity at site A21 together with computed curves from best-fit five-layer (dashed curves) and smooth 1D (solid curves) inversion models of Supplementary Figure 11.

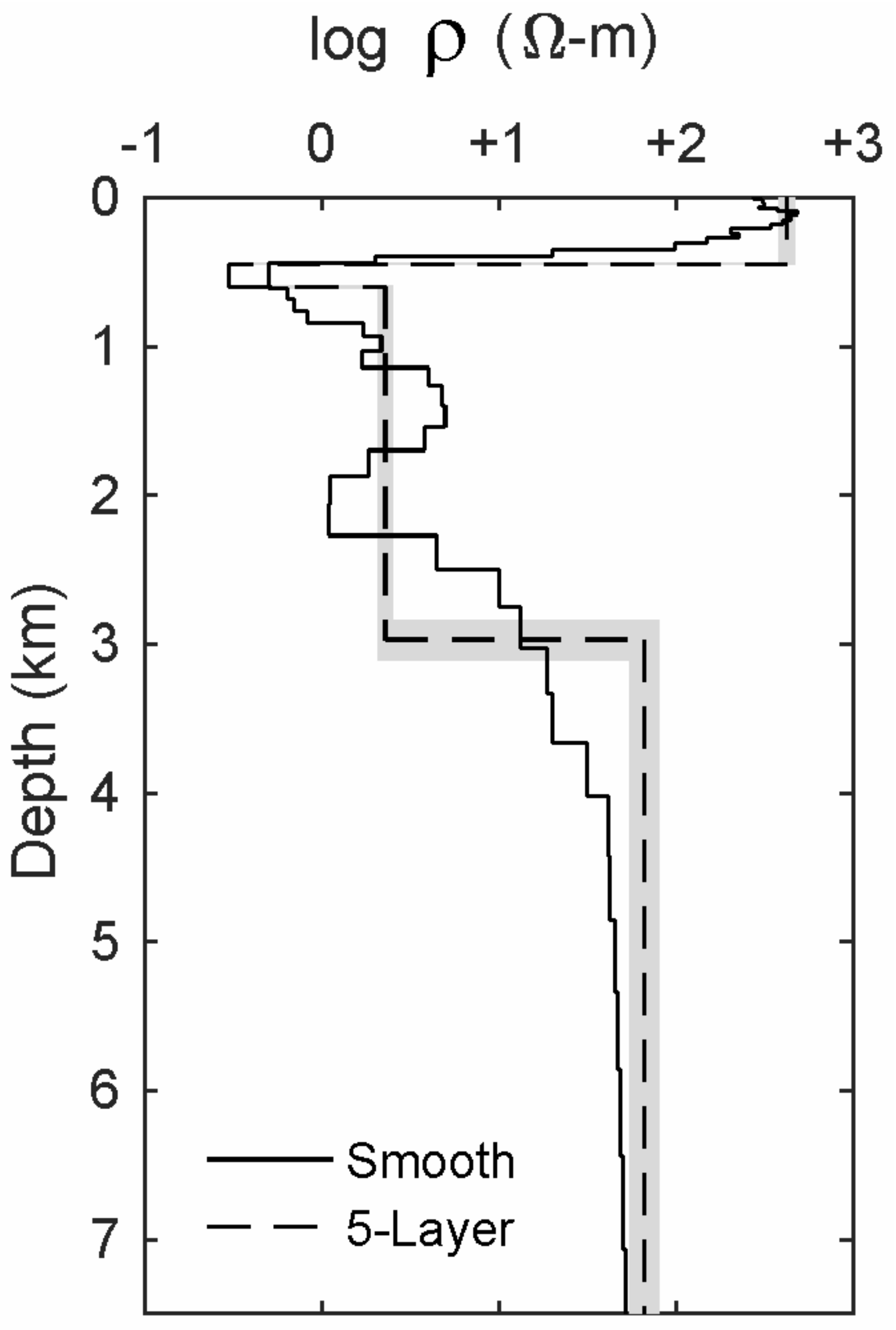

Supplementary Figure 11

Best-fit five-layer (dashed lines) and smooth 1D (solid lines) inversion models for site A21 of Supplementary Figure 10 on the RIS. Gray bars of five-layer model denote one standard deviation spread in parameter estimates. Seawater layer fixed at 0.3 ohm-m for five-layer model.

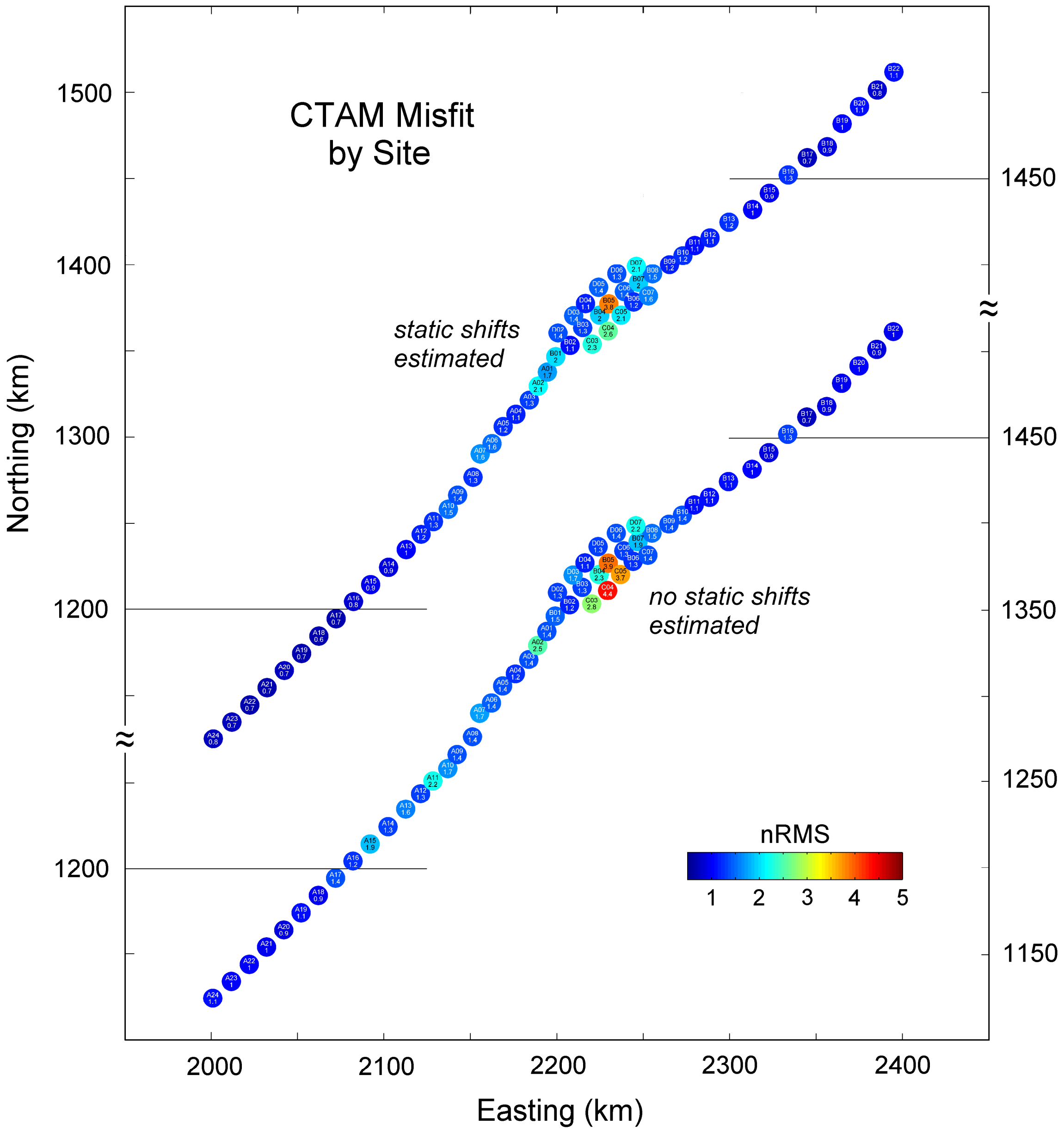

Supplementary Figure 12

Normalized root-mean-square (nRMS) misfits on a site-to-site basis for the CTAM data set compared for the inversion model with (Fig. 6) and without (Supplementary Figure 11) impedance static distortions estimated. Note that only a few sites dominate the misfit before static estimation.

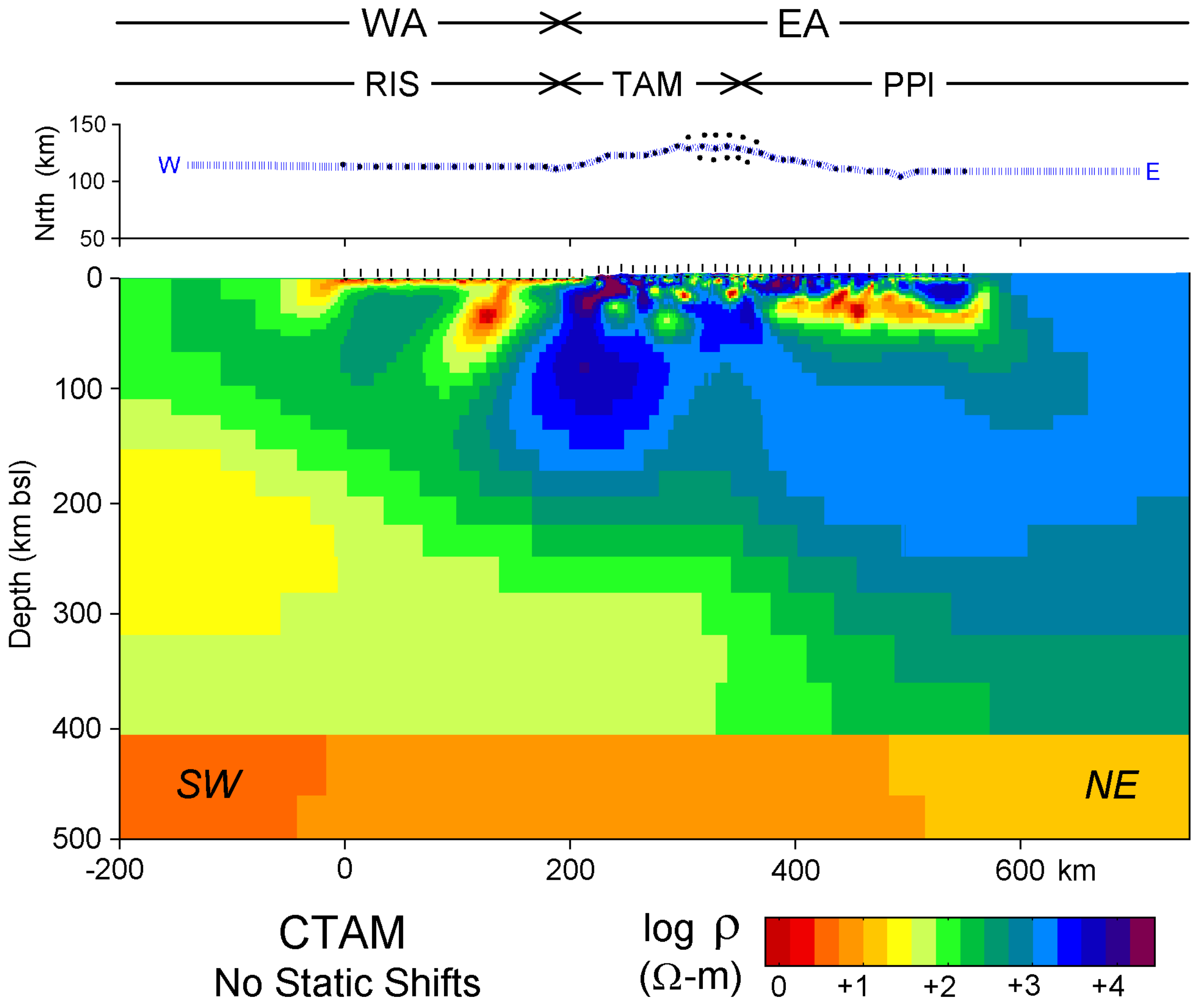

Supplementary Figure 13

Section view of 3D CTAM inversion model computed without impedance static distortion estimation. Conventions otherwise as in Fig. 6.

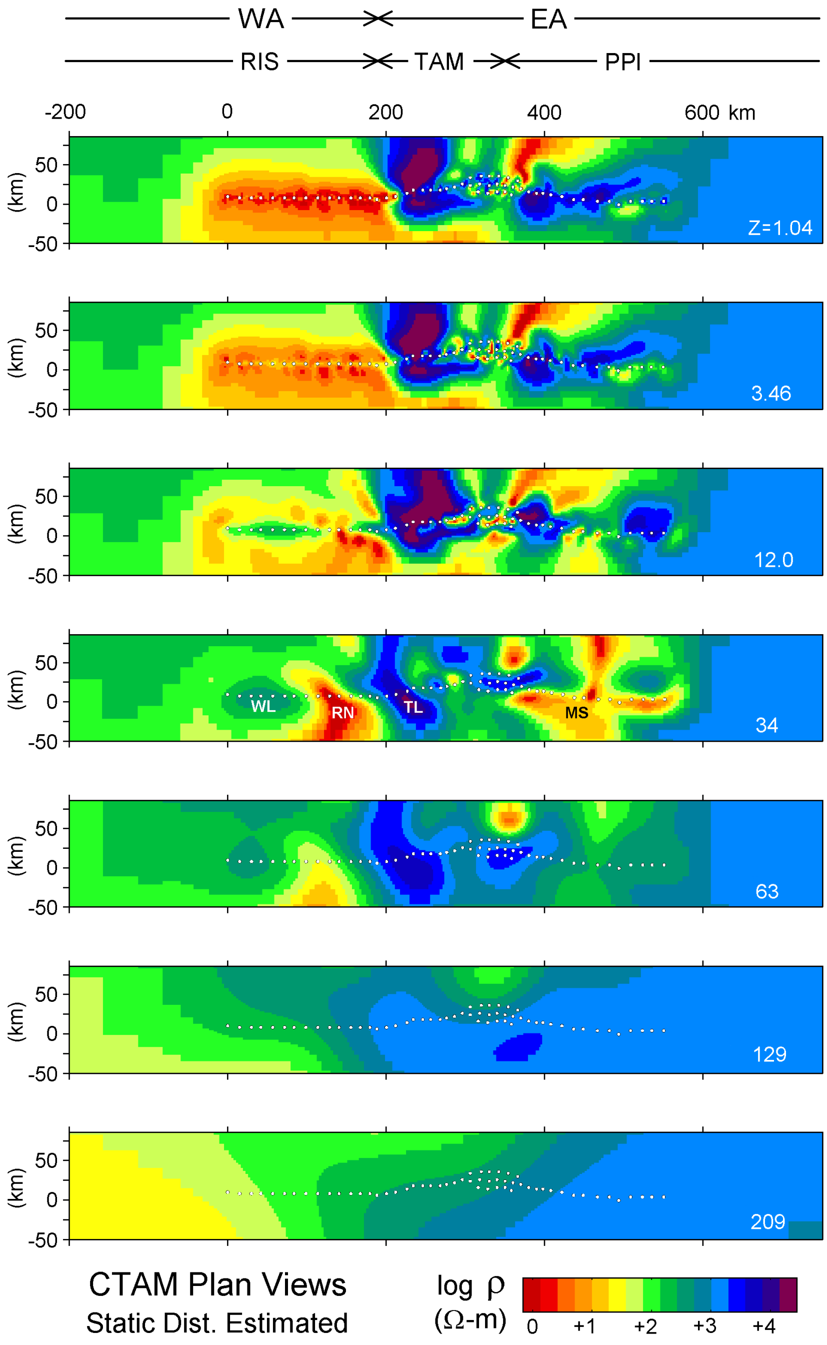

Supplementary Figure 14

Plan views at seven depth levels (in km) for the CTAM MT inversion model computed with impedance static distortion estimation.

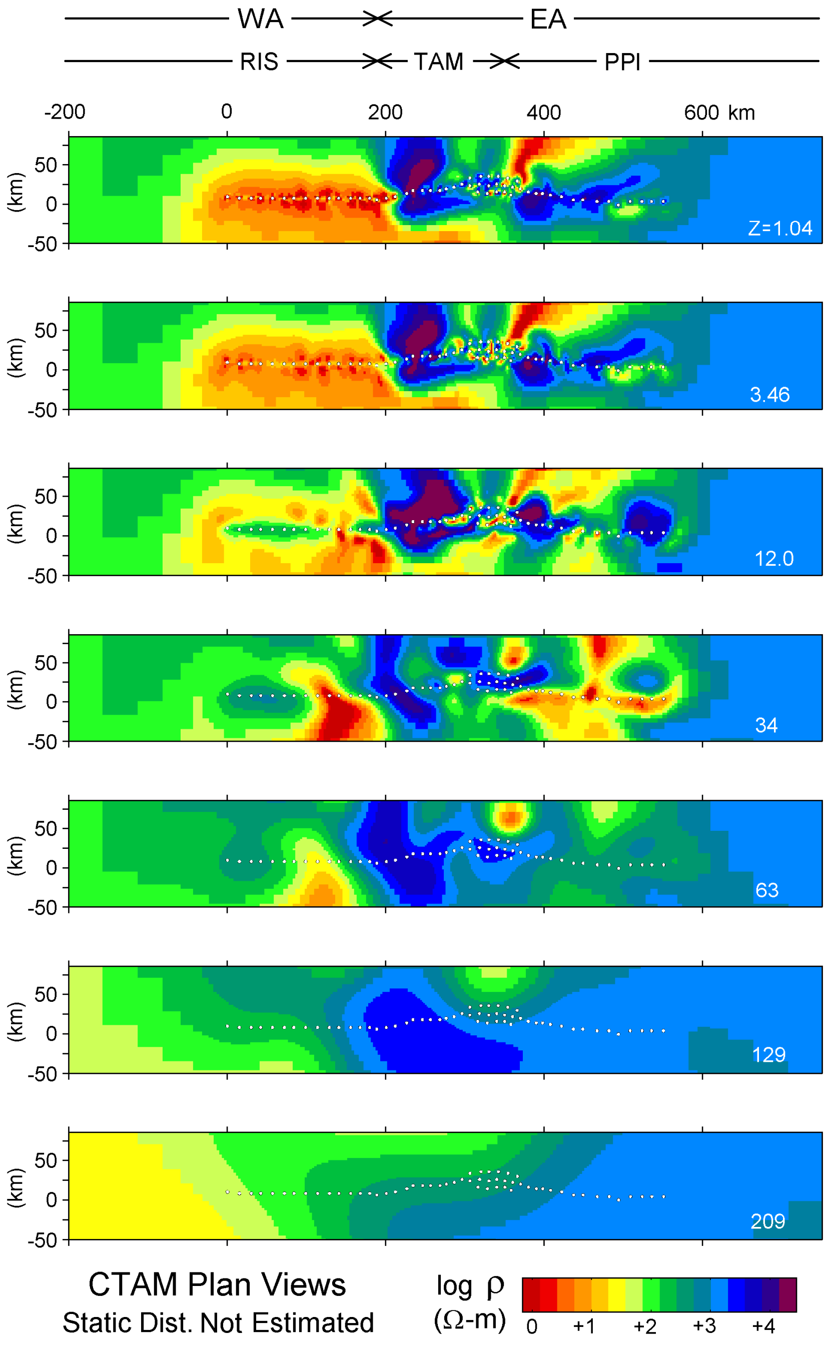

Supplementary Figure 15

Plan views at seven depth levels (in km) for the CTAM MT inversion model computed without impedance static distortion estimation.

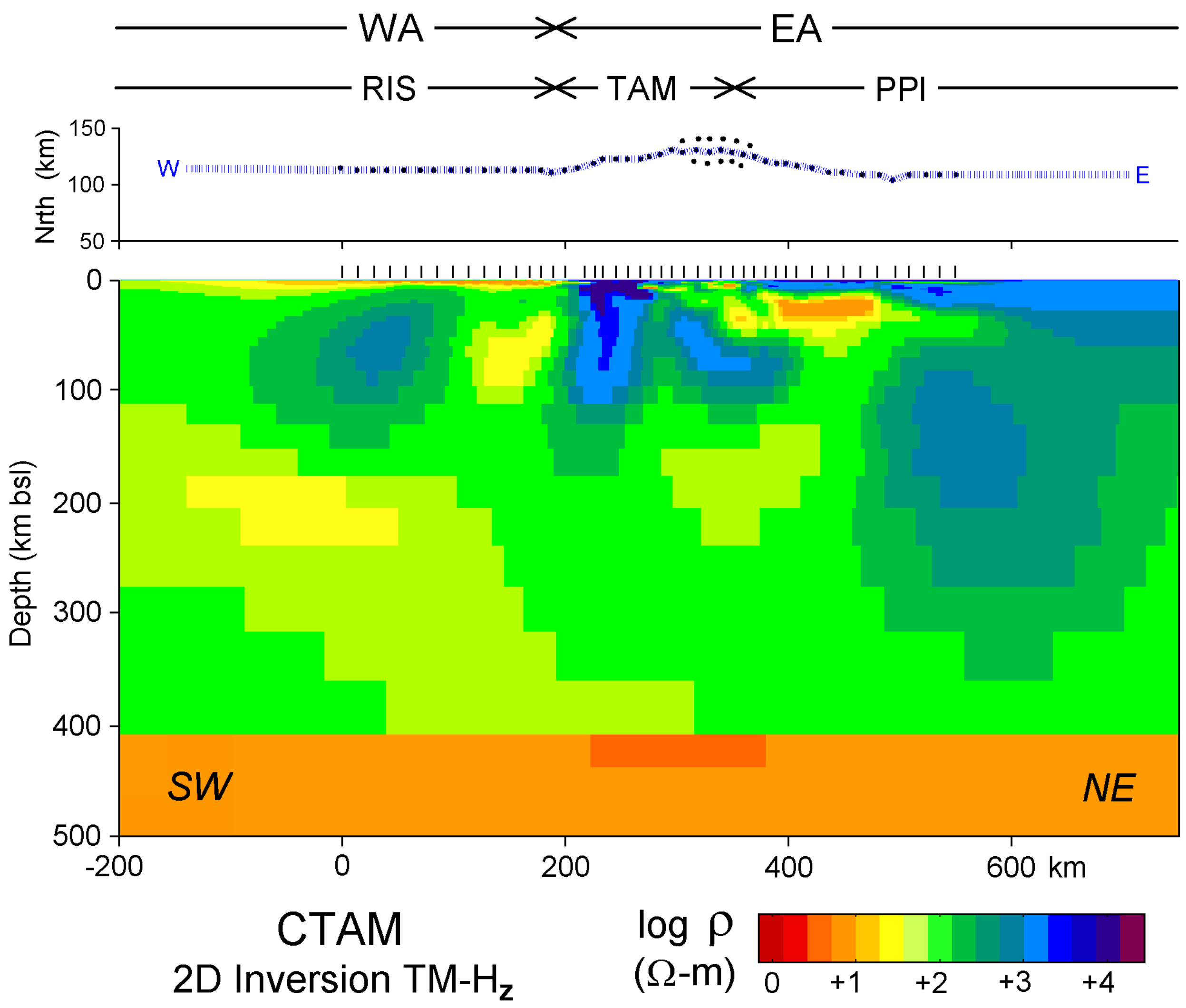

Supplementary Figure 16

Two-dimensional inversion model of CTAM transect utilizing nominal TM (yx) mode impedance and tipper element K_{zy}. Computed model response shown in Fig. 4.

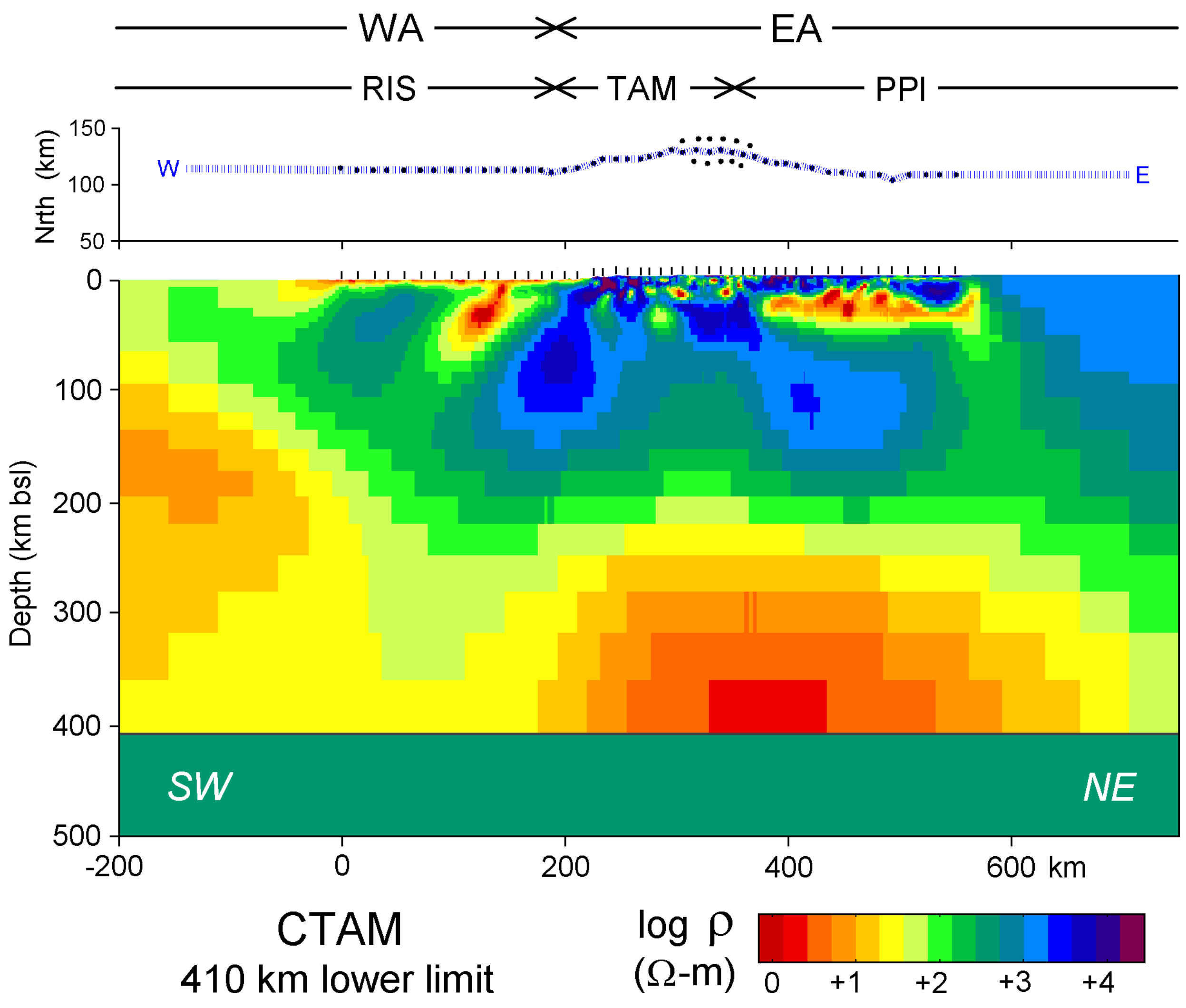

Supplementary Figure 17

Three-dimensional inversion model of CTAM MT transect where resistivity below 410 km depth was set to 300 ohm-m and not allowed to vary across the iterations.

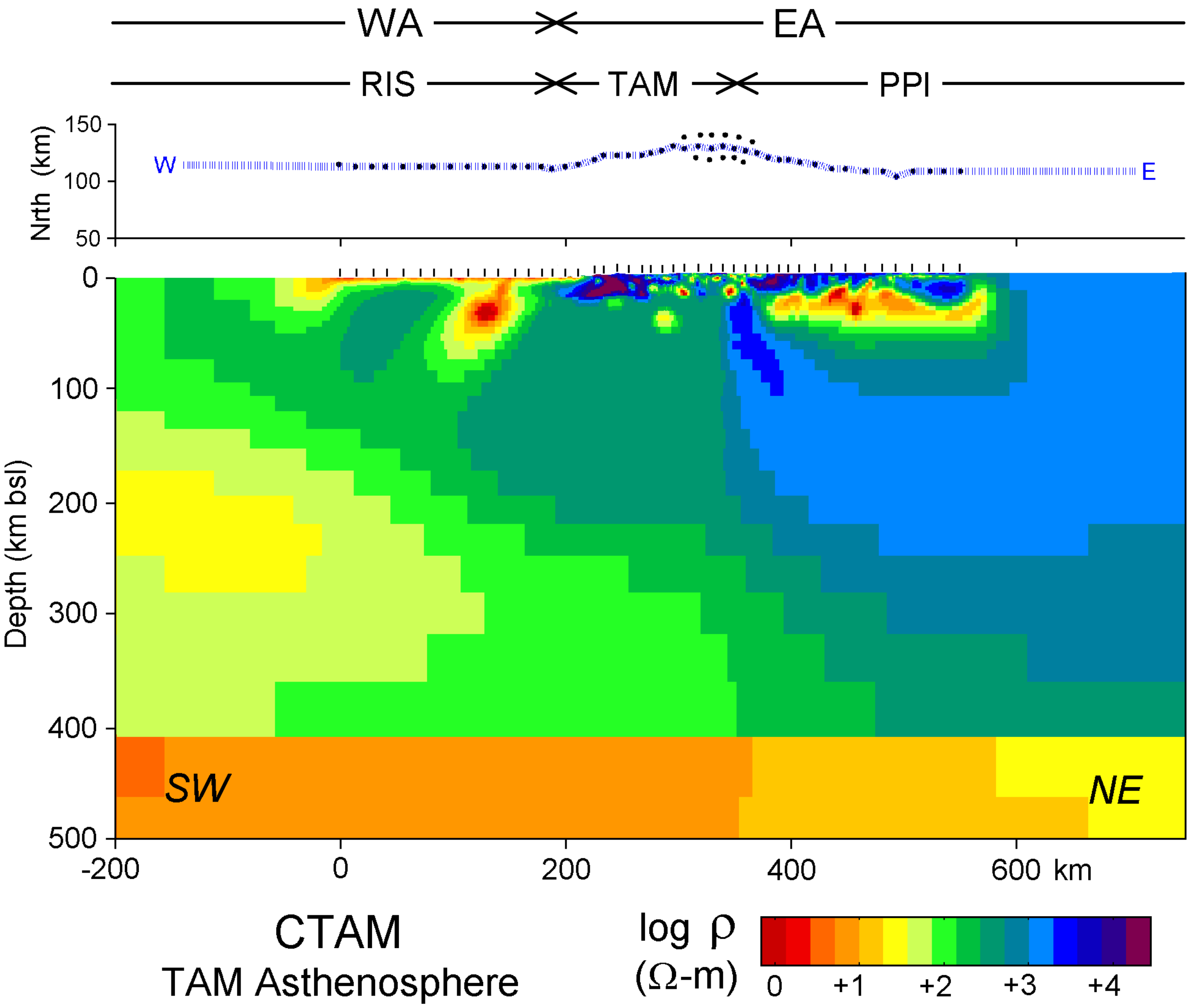

Supplementary Figure 18

Preferred model of Fig. 6 with RIS lithospheric resistivity properties artificially extended beneath the TAM to the PPl transition to assess misfit to observations. nRMS misfit by site shown in Supplementary Figure 19.

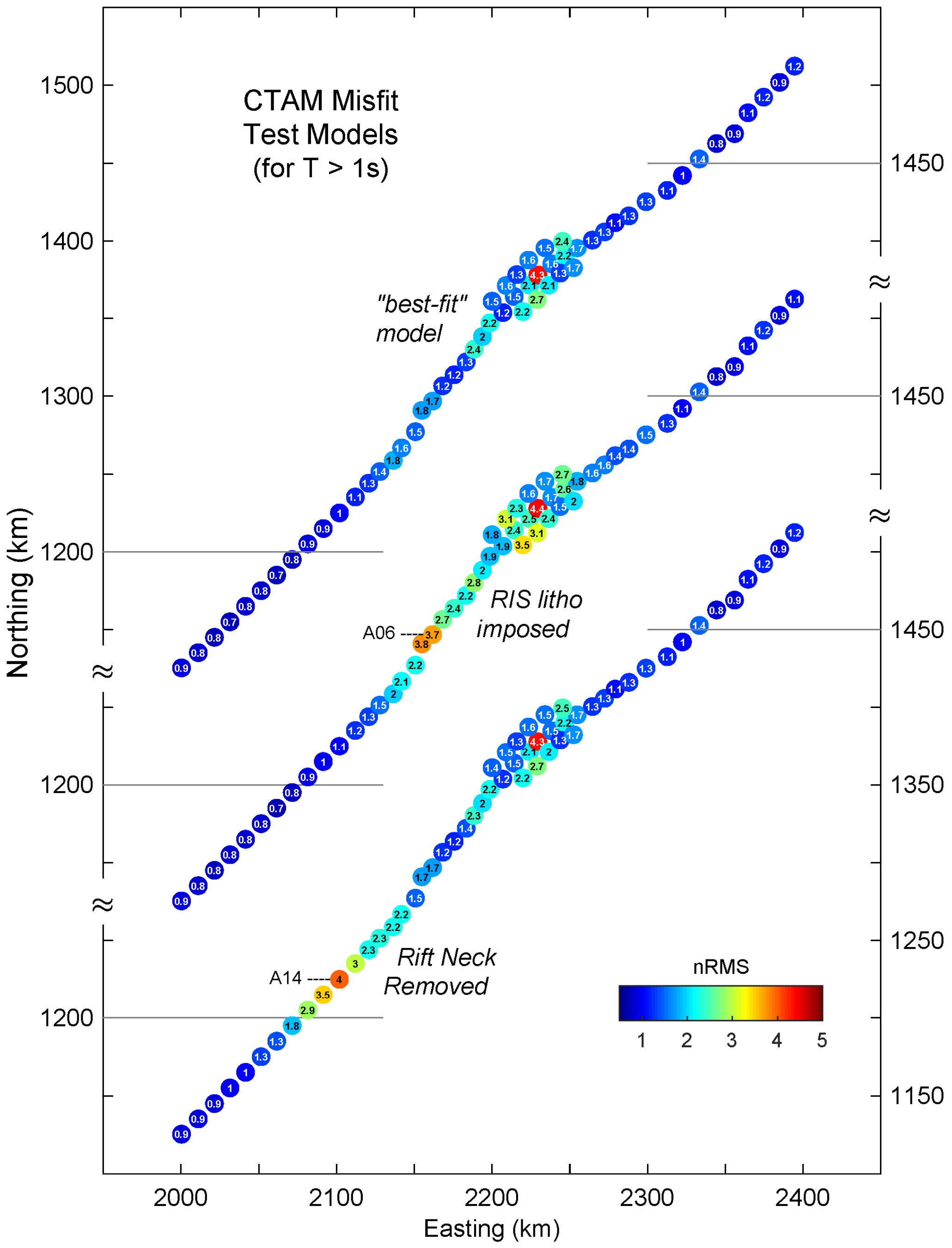

Supplementary Figure 19

Normalized root-mean-square (nRMS) misfits on a site-to-site basis for the CTAM data set for the preferred inversion model (top), for the test model with RIS lithospheric resistivity properties artificially extended beneath the TAM to the PPl transition (Supplementary Figure 16) (middle), and for the test model with conductive rift neck structure at the RIS-TAM transition artificially removed (Supplementary Figure 20) (bottom). Sites A06 and A14 are denoted for location in Fig. 1.

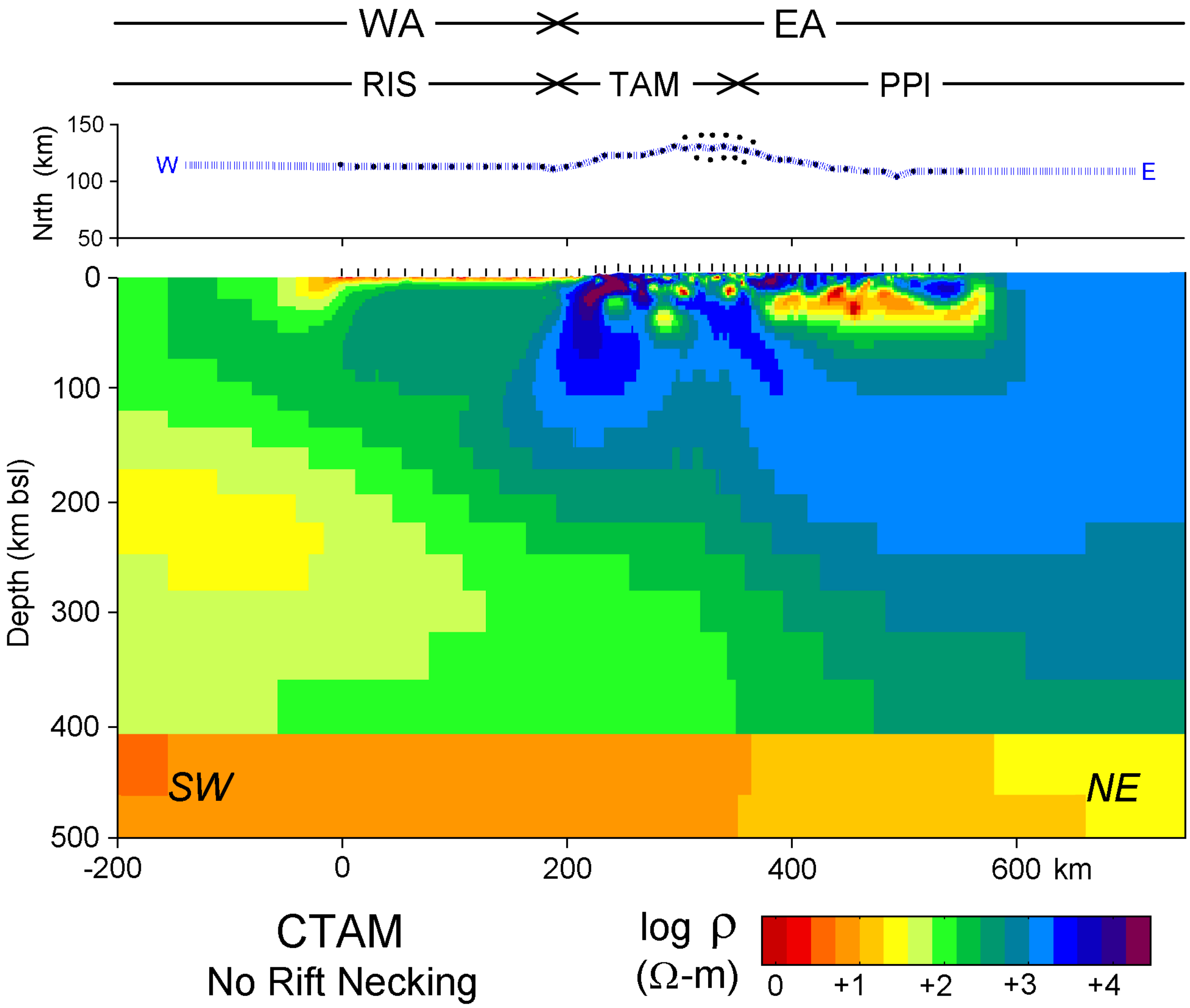

Supplementary Figure 20

Preferred model of Fig. 6 with conductive rift neck structure at the RIS-TAM transition artificially removed to assess misfit to observations. nRMS misfit by site shown in Supplementary Figure 19.
